# Supplementary material for: Effect of inverted visual acceleration profile on vestibular heading perception
Source: PLoS One. 2025 May 28;20(5):e0323348. doi: 10.1371/journal.pone.0323348 (PMC12118926; doi:10.1371/journal.pone.0323348)
Supplement: Attachment — Submitted filename: Comments.docx [file pone.0323348.s003.docx]

This study investigates the integration of visual and inertial cues in influencing heading perception during motion. The authors found that inverted visual stimuli consistently biased inertial heading perception less than matched velocity stimuli across different offsets. This represents a novel and valuable contribution to the field of sensory integration and perception. The manuscript is generally well-organized, but I have a few specific comments listed below:

**Abstract:**

There is a spelling mistake for “whish,” which should be corrected.

**Introduction:**

It would be beneficial to provide more explanation for the choice of large offset values (±45°, ±60°, ±75°). Subjects might use the discrepancy between visual and vestibular cues to infer their causal relationship (refer to Acerbi L, Dokka K, Angelaki DE, Ma WJ, 2018. Bayesian comparison of explicit and implicit causal inference strategies in multisensory heading perception. PLoS Comput Biol 14(7): e1006110).

Numbers and units are sometimes separated by spaces, and sometimes they are not, which creates inconsistency, for example, '2s' and '2 s'.

**Results:** More detailed descriptions of the figures within the text are needed, explaining what each figure shows and its significance.

The variation between subjects is noted but not explored in depth. Potential reasons for individual differences in bias should be discussed, including how these differences might affect the overall results.

**Discussion:**

The interpretation of the results is somewhat superficial. A deeper analysis of the implications of the findings and their advancement of the field is needed.

Provide a more in-depth interpretation of the results, considering alternative explanations and the broader implications.

**Bibliography:**

Several instances of “Deangelis” are misspelled and should be corrected to “DeAngelis.”
