# Supplementary material for: Effect of inverted visual acceleration profile on vestibular heading perception
Source: PLoS One. 2025 May 28;20(5):e0323348. doi: 10.1371/journal.pone.0323348 (PMC12118926; doi:10.1371/journal.pone.0323348)
Supplement: Attachment — Submitted filename: Response to reviewers.docx [file pone.0323348.s007.docx]

After assessing your revisions, I feel that Reviewer #4's comment regarding the ANOVA design has not been fully resolved. Reviewer #4 rightly pointed out that for a 3x2 repeated-measures ANOVA design with n=20 participants, the degrees of freedom for the two-level factor main effect are F(1,19), and for the three-level factor main effect as well as for the interaction effect the df are F(2,38). Given that you report F(1,2) as the df of the two-way ANOVA, it appears that statistics were not properly run on individual means. Moreover, reporting of ANOVA and t-test results still does not follow the conventional form pointed out by Reviewer #4 (i.e., "F(df1,df2) = F-value, p = p-value" for each ANOVA effect and "t(df) = t-value, p = p-value" for each t-test). Please address these issues with your next revision.

To review here are the previous comments of reviewer #4:

Reviewer #4: The authors have addressed most of my comments but I just want to clarify the ANOVA and how it has been reported. It is a bit confusing as in the analysis the ANOVA is given as an offset x stimulus 3(45 deg, 60 deg and 70 deg)x2(normal and inverted). But in the results it seems to be 6x1 (45 deg x normal, 60 deg x normal, 70 deg x normal, 45 deg x inverted, 60 deg x inverted and 70 deg x inverted). If it is a 2x3 the DoF should be: for the stimulus 1,19 for the offset 2,38 for the interaction 2,38 If it is a 6x1 then the DoF is 5 but it does not test the offset and stimulus separately, which means was a followup ANOVA conducted. Can the authors clarify this. What I would suggest is for the ANOVAs report the results as F(df1,df2)=?,p< and for the t-tests state t(df)=??,p

**Response: We have made the proposed changes:**

**The first paragraph of the results now states:**

**A three-way ANOVA was performed using visual stimulus type (normal, inverted) and the three non-zero offsets (45, 60, 75°). This (ANOVA, F(1,38) = 9.61) revealed that visual stimulus type is the only statistically significant factor (p = 0.0022) with no effect of offset magnitude (F(2, 38) = 0.28, p = 0.76). There was a significant effect between subjects (ANOVA, F(6,19) = 13.2, p < 0.0001) A paired T-test of inverted vs. normal visual stimulus was statistically significant t(118) = 6.487, p < 0.0001 (two-tailed) when all the non-zero offsets were considered. When the offsets were considered separately, the effect remained statistically significant at 45° t(39) = 3.04, p = 0.004, 60° t(39) = 4.18, p = 0.0002, and 75° t(39) = 4.58, p < 0.0001.**

**The third paragraph is also changed:**

**When the potential effects of visual stimulus type and offset were examined there were no statistically significant effects using a three-way ANOVA to look at stimulus type and non-zero offsets. When the visual stimulus profile was examined (ANOVA, F(1,38) = 0.36) revealed that the stimulus type (p = 0.55) was not a significant factor. When offset magnitude was examined (ANOVA. F(2,38) = 0.20) there was also no significant effect (p = 0.82). As with the bias, there was significant variation in sigma between subjects (ANOVA, F(6,19) = 3.38, p < 0.0001). Overall, the normal stimulus had a sigma of 6.1 ± 0.4 (SEM) and the inverted stimulus had a sigma of 5.6 ± 0.4 (SEM), but this difference was not statistically significant t(179) = -0.94, p = 0.35.**
