# Supplementary material for: Effect of inverted visual acceleration profile on vestibular heading perception
Source: PLoS One. 2025 May 28;20(5):e0323348. doi: 10.1371/journal.pone.0323348 (PMC12118926; doi:10.1371/journal.pone.0323348)
Supplement: Attachment — Submitted filename: Complete reviewer comments 2.docx [file pone.0323348.s005.docx]

CC: [patrick.bruns@uni-hamburg.de](mailto:patrick.bruns@uni-hamburg.de)

PONE-D-24-23927

Effect of Inverted Visual Acceleration Profile on Vestibular Heading Perception.

PLOS ONE

Dear Dr. Crane,

Thank you for submitting your manuscript to PLOS ONE. After careful consideration, we feel that it has merit but does not fully meet PLOS ONE’s publication criteria as it currently stands. Therefore, we invite you to submit a revised version of the manuscript that addresses the points raised during the review process.

As you will see below, while the expert reviewers were mainly positive about your work, they also raised some concerns, most notably the large between-subject variability (Reviews #1 and #2), the lack of unimodal baseline conditions and eye-tracking recordings (Review #3), and potential differences in the reliability of the responses between the two visual motion profiles (Review #4). Please address these concerns in your revision of the manuscript. I will likely send out the new version to some or all of the previous reviewers.

Please submit your revised manuscript by Sep 14 2024 11:59PM. If you will need more time than this to complete your revisions, please reply to this message or contact the journal office at [plosone@plos.org](mailto:plosone@plos.org). When you're ready to submit your revision, log on to <https://www.editorialmanager.com/pone/> and select the 'Submissions Needing Revision' folder to locate your manuscript file.

Please include the following items when submitting your revised manuscript:

- A rebuttal letter that responds to each point raised by the academic editor and reviewer(s). You should upload this letter as a separate file labeled 'Response to Reviewers'.
- A marked-up copy of your manuscript that highlights changes made to the original version. You should upload this as a separate file labeled 'Revised Manuscript with Track Changes'.
- An unmarked version of your revised paper without tracked changes. You should upload this as a separate file labeled 'Manuscript'.

If you would like to make changes to your financial disclosure, please include your updated statement in your cover letter. Guidelines for resubmitting your figure files are available below the reviewer comments at the end of this letter.

If applicable, we recommend that you deposit your laboratory protocols in [protocols.io](http://protocols.io) to enhance the reproducibility of your results. [Protocols.io](http://Protocols.io) assigns your protocol its own identifier (DOI) so that it can be cited independently in the future. For instructions see: <https://journals.plos.org/plosone/s/submission-guidelines#loc-laboratory-protocols>. Additionally, PLOS ONE offers an option for publishing peer-reviewed Lab Protocol articles, which describe protocols hosted on [protocols.io](http://protocols.io). Read more information on sharing protocols at <https://plos.org/protocols?utm_medium=editorial-email&utm_source=authorletters&utm_campaign=protocols>.

We look forward to receiving your revised manuscript.

Kind regards,

Patrick Bruns
Academic Editor
PLOS ONE

Journal Requirements:

When submitting your revision, we need you to address these additional requirements.

1. Please ensure that your manuscript meets PLOS ONE's style requirements, including those for file naming. The PLOS ONE style templates can be found at 
<https://journals.plos.org/plosone/s/file?id=wjVg/PLOSOne_formatting_sample_main_body.pdf> and 
<https://journals.plos.org/plosone/s/file?id=ba62/PLOSOne_formatting_sample_title_authors_affiliations.pdf>

2. Thank you for stating the following financial disclosure: "Grant support was provided by 2 R01 DC013580. EA was supported in part by the National Institutes of Health (NIDCD K23 DC018303)"  
Please state what role the funders took in the study.  If the funders had no role, please state: ""The funders had no role in study design, data collection and analysis, decision to publish, or preparation of the manuscript."" 
If this statement is not correct you must amend it as needed. 
Please include this amended Role of Funder statement in your cover letter; we will change the online submission form on your behalf.

3. Thank you for stating the following in the Acknowledgments Section of your manuscript: "The authors would like to thank Cesar Arduino for providing technical assistance for the experiments. Grant support was provided by 2 R01 DC013580. EA was supported in part by the National Institutes of Health (NIDCD K23 DC018303).
Manuscript Click here to access/download;Manuscript;BTC Vis Accel PLoS
v2.docx Yakouma, et al. Inverted Visual and Vestibular Heading Perception"
We note that you have provided funding information that is not currently declared in your Funding Statement. However, funding information should not appear in the Acknowledgments section or other areas of your manuscript. We will only publish funding information present in the Funding Statement section of the online submission form. 
Please remove any funding-related text from the manuscript and let us know how you would like to update your Funding Statement. Currently, your Funding Statement reads as follows: "Grant support was provided by 2 R01 DC013580. EA was supported in part by the National Institutes of Health (NIDCD K23 DC018303)"

The funding was removed from the acknowledgments. The funding statement has been updated.

Please include your amended statements within your cover letter; we will change the online submission form on your behalf.

[Note: HTML markup is below. Please do not edit.]

Reviewers' comments:

Reviewer's Responses to Questions

**Comments to the Author**

1. Is the manuscript technically sound, and do the data support the conclusions?

The manuscript must describe a technically sound piece of scientific research with data that supports the conclusions. Experiments must have been conducted rigorously, with appropriate controls, replication, and sample sizes. The conclusions must be drawn appropriately based on the data presented.

Reviewer #1: Yes

Reviewer #2: Yes

Reviewer #3: Yes

Reviewer #4: Partly

2. Has the statistical analysis been performed appropriately and rigorously?

Reviewer #1: Yes

Reviewer #2: Yes

Reviewer #3: Yes

Reviewer #4: No

3. Have the authors made all data underlying the findings in their manuscript fully available?

The [PLOS Data policy](http://www.plosone.org/static/policies.action#sharing) requires authors to make all data underlying the findings described in their manuscript fully available without restriction, with rare exception (please refer to the Data Availability Statement in the manuscript PDF file). The data should be provided as part of the manuscript or its supporting information, or deposited to a public repository. For example, in addition to summary statistics, the data points behind means, medians and variance measures should be available. If there are restrictions on publicly sharing data—e.g. participant privacy or use of data from a third party—those must be specified.

Reviewer #1: Yes

Reviewer #2: Yes

Reviewer #3: Yes

Reviewer #4: No

4. Is the manuscript presented in an intelligible fashion and written in standard English?

PLOS ONE does not copyedit accepted manuscripts, so the language in submitted articles must be clear, correct, and unambiguous. Any typographical or grammatical errors should be corrected at revision, so please note any specific errors here.

Reviewer #1: Yes

Reviewer #2: Yes

Reviewer #3: Yes

Reviewer #4: Yes

5. Review Comments to the Author

Please use the space provided to explain your answers to the questions above. You may also include additional comments for the author, including concerns about dual publication, research ethics, or publication ethics. (Please upload your review as an attachment if it exceeds 20,000 characters)

Reviewer #1: In the current study, the author presented visual stimuli with different temporal profile, namely, inverted, compared to vestibular, and found that it generated less impact on the the combined performance. The results are clear. The manuscript is well written. I do not have many comments. The only query is that although the results are quite clear for the mean PSE shift comparison between inverted and normal visual stimulation condition, yet they are quite varied for individual subjects (figure 4).

Thanks. We agree that there was a lot of individual variation. In our experience, this is fairly typical of these types of human psychophysics experiments.

Reviewer #2: This study investigates the integration of visual and inertial cues in influencing heading perception during motion. The authors found that inverted visual stimuli consistently biased inertial heading perception less than matched velocity stimuli across different offsets. This represents a novel and valuable contribution to the field of sensory integration and perception. The manuscript is generally well-organized, but I have a few specific comments listed below:

Thanks. We agree.

Abstract:
There is a spelling mistake for “whish,” which should be corrected.

This was corrected to “which”

Introduction:
It would be beneficial to provide more explanation for the choice of large offset values (±45°, ±60°, ±75°). Subjects might use the discrepancy between visual and vestibular cues to infer their causal relationship (refer to Acerbi L, Dokka K, Angelaki DE, Ma WJ, 2018. Bayesian comparison of explicit and implicit causal inference strategies in multisensory heading perception. PLoS Comput Biol 14(7): e1006110).

Thanks. The Acerbi et al paper used smaller offsets (5, 10, 20, and 40°). The offsets we used were based on previous studies in which visual stimuli could bias the vestibular heading direction with offsets up to 90° [Rodriguez, 2019 #2059] even though beyond about 30° subjects were aware these stimuli did not have a causal relationship [Rodriguez, 2020 #2084]. In the current study, we weren’t interested in determining if subjects inferred a causal relationship because the lack of a cause relationship would have been obvious from the inverted velocity profile of the visual stimulus. We now make this clear in the introduction:

“The purpose of this study was not to look at causal inference[18] as it would have been obvious to most observers that the inverted visual stimulus was inconsistent with the inertial motion. We had previously observed that visual stimuli bias inertial direction perception even with large offsets of up to 90° when it was clear to subjects that the stimuli were artificially offset[5]. Thus, the range of offsets used in the current study went to 75°.”

Numbers and units are sometimes separated by spaces, and sometimes they are not, which creates inconsistency, for example, '2s' and '2 s'.

Thanks for noticing this. We have now standardized to not have a space between the number and the s (e.g. 2s).

Results: More detailed descriptions of the figures within the text are needed, explaining what each figure shows and its significance.

Thanks. We now explain in detail each panel for Figure 1:

“The inverted visual stimulus also lasted 2s (Fig. 1) and had a similar peak acceleration (Fig. 1A), peak velocity (Fig. 1B), and displacement (Fig. 1C. The difference between the normal and inverted visual stimulus was that the inverted stimulus began and ended at the peak velocity while the normal stimulus achieved the peak velocity at the midpoint (Fig. 1B). However, both stimuli were consistent with the same displacement, and had the same peak velocity and peak acceleration.”

For figure 2, the details of the figure are explained in more detail in the Analysis section:

“The fraction of rightward responses for each stimulus level was plotted as a function of the heading direction tested for both the normal velocity (Fig. 2A-C) and inverted velocity (Fig. 2 D-F). The PSE was determined by fitting a cumulative distribution function using the fit function in Matlab (version R2019b) for data collected from otherwise similar staircases that started in opposite directions (Fig. 2). This determined the mean of the psychometric function (also the PSE) at which responses were equally likely to be reported in each direction. The width of the psychometric function (i.e. sigma or standard deviation of the psychometric function) was also determined. In the example shown, offsetting the normal visual stimulus to the left (Fig. 2A) tended to bias the perception of inertial direction such that an inertial stimulus had to be shifted 10.3° to the right to be perceived as neutral (i.e. straight ahead). The opposite bias was seen when the visual stimulus was shifted towards the right (Fig. 2C). Visual offsets produced smaller biases with the inverted velocity stimulus (Figs. 2D and 2F).”

Figure 3 is a simple figure that is explained at the start of the Results. It is now qualified that this shows the aggregate data.

Figure 4 is now described in greater detail in the text. At the end of the first paragraph of the results it now says: “Given that the size of the offset did not have a significant effect, the non-zero offsets were combined to show the individual data so that the effect of the visual stimulus could be seen for each of the twenty subjects (Fig. 4).”

The variation between subjects is noted but not explored in depth. Potential reasons for individual differences in bias should be discussed, including how these differences might affect the overall results.

Other reviewers raised similar criticisms. The variation is now better described in the results as well as the discussion.

Discussion:
The interpretation of the results is somewhat superficial. A deeper analysis of the implications of the findings and their advancement of the field is needed.

Provide a more in-depth interpretation of the results, considering alternative explanations and the broader implications.

Thanks, similar comments were also made by other reviewers, and this is now included. We now discuss the variation between subjects and have also presented and discussed the width of the psychometric fits (sigma). We also now discuss the possibility that the reliability of the normal and inverted visual stimuli may have caused them to be perceived differently. The observation that the bias did not depend on the size of the offset is also now discussed.

Bibliography:
Several instances of “Deangelis” are misspelled and should be corrected to “DeAngelis.”

Thanks for noticing this. It has been corrected to “DeAngelis”

Reviewer #3: The authors present the results of a psychophysical study on heading perception of n=20 healthy participants while they experienced 2 s episodes of passive inertial self motion (15 cm displacements on a 6DoF motion platform, see Fig. 1). Participants viewed random-dot kinematic (RDK) noise fields that simulated congruent (in phase with platform motion) or incongruent (out-of-phase with platform motion) optic flow sequences. The focus of expansion (FoE) of the flow fields was offset +/- 45, 60, or 75 degrees. The hypotheses test the effects of phase differences between visual (RDKs) and vestibular (self motion on platform) and direction offsets on the subjective point of straight ahead self motion. The results indicate that the direction offset of the FoE of RDK visual motion biases estimates of straight ahead self motion in participants who viewed in-phase (congruent) visual RDK flow fields more compared to conditions where the velocity profile of the RDK flow fields was out of phase to the platform motion (Fig. 3). The results are discussed in terms of multisensory integration of visual and vestibular cues of self motion.

The work appears to have been carefully conducted. Some clarifications are needed (see below).

Thanks

Major revision requests

1) It seems that some control conditions could have been included to determine the extent that the participants were truly integrating visual and vestibular cues of self motion. The most obvious ones would be to place the participants blindfolded on the motion platform to determine their acuity of sensing "straight ahead" self motion without visual input. In the same manner, the participants could have been asked to judge the FoE of RDK flow fields (left or right of straight ahead) in the complete absence of platform motion. The results of these control conditions would have provided a "baseline" for direction judgments about these visual and vestibular stimuli.

We have done and published these types of control experiments previously, although in a different subject panel as a part of unrelated experiments[Crane, 2017 #1802][Rodriguez, 2018 #1964][Crane, 2012 #1280]. What previous work on this has shown is that subjects are able to judge visual headings with a high degree of accuracy and precision. Inertial headings in darkness were less precise and also would often have a small bias.

In the current study, subjects were asked to judge only the inertial direction. We felt it would be confusing to include conditions where no inertial stimulus was present and visual headings were reported as this might encourage subjects to report the visual heading in subsequent experiments. Furthermore, the reliability of the visual stimulus has been shown to decrease as the heading gets further from straight ahead. Obtaining unisensory control data for the range of visual headings included would have involved another series of experiments that would have greatly complicated the data collection and wasn’t done.

In the current experiments, the control condition was when the visual heading was not offset, and this provides the “baseline” that the reviewer requests. We understand that the performance of an inertial heading in darkness may be different than when a visual heading was available.

At this point, we no longer have access to many of the subjects in this panel as it is not possible to do additional control experiments.

The following has been added to the discussion:

“The current experiments did not include any unisensory conditions (I,e. visual only or initial only) although such experiments have been previously published in our laboratory[Crane, 2017 #1802][Rodriguez, 2018 #1964][Crane, 2017 #1802]. We did not want to include a visual-only condition because subjects were specifically asked to identify the inertial heading, and it would likely be confusing. Although an inertial-only condition could have quantified underlying biases, these were ultimately cancelled by averaging the effects of visual offsets in opposite directions.”

2) There is no mention of eye-movement recordings during the different visual-vestibular stimulus conditions. The stimulus displays depicted in Fig. 2 show no indication of any stationary fixation marks in the RDK flow fields. Were the participants instructed to direct their gaze "straight ahead" and if so how was compliance monitored during trials with platform motions? Participants would naturally direct their gaze to the focus of expansion in flow fields. Did the authors note such behaviour and if so how did it influence their judgments about straight ahead with respect to their own self motion?

Eye movements were not recorded in this study. No fixation point was provided, and participants were not given any instructions on where to look. We have previously published data where eye position was carefully monitored and controlled. Subjects were not given any instruction on gaze position. This is now clarified in the Methods: “No fixation point was provided, and subjects were not given any specific instructions on where to look.”

We have previously done experiments to examine the effect of gaze position [Rodriguez, 2018 #1964][Crane, 2012 #1280]. As the reviewer mentions it is difficult to maintain gaze at a particular location without a fixation point and when a fixation point is present, subjects tend to judge heading as the location of the focus of expansion relative to the fixation point. Subjects were asked to report only inertial heading in these experiments and this perception is in body coordinates and insensitive to eye position. Of course, it is possible that eye position had some effect. We added a paragraph to address this concern:

“In the current study, there were no attempts to control or measure gaze position. Gaze position was controlled and measured in some previous studies in the current laboratory[Rodriguez, 2018 #1964][Crane, 2012 #1280]. This was not done in the current study because it is difficult to maintain a gaze position without a fixation point and when a fixation point is present subjects tend to judge the heading by reporting the focus expansion relative to the fixation point. Subjects were instructed to report only the direction of the inertial stimulus which has previously been demonstrated to be independent of eye position[Crane, 2012 #1280][Cuturi, 2013 #1412]. Thus, the effect of gaze position in the current paradigm is unknown but likely minimal.”

3) The data from participant #17 should be removed as a statistical outlier and the data analysis should be performed without these outlier data. It is unclear why P#17 was perceiving inertial self motion at +/-45 degrees as "straight ahead" more or less independent of the visual motion cues.

We agree that subject #17 is an outlier. Our interpretation of this data is that the visual stimulus had a strong influence on this subject’s perception. Another possible interpretation of this could be that this subject ignored the inertial stimulus and reported only the visual stimulus direction. However, the effect of the inverse visual stimulus was still less than the normal visual stimulus, so it does not appear the inertial stimulus was ignored. If this subject were excluded the overall conclusions of the paper would be the same. If one were to exclude outliers an argument could also be made to exclude subjects #1 and #8 who essentially had the opposite effect in that only the inertial stimulus was reported. Overall, it is the author's view that all data should be included and presented, if a reader feels some data shouldn’t be considered they are free to make this choice. We added a paragraph to the Discussion to address this:

“There was significant variation between subjects. Subject #17 was an outlier in that their bias towards visual stimulus was much larger than any of the other subjects (Fig. 4). An argument could be made for excluding this subject as a statistical outlier but, we chose to leave it in. The subject was an 18-year-old white male, so he was not an outlier in terms of demographic. The sigma for this subject (Fig. 5) was not an outlier. One possible interpretation of this subject’s response was that they did not rigorously follow the instructions and reported the direction of the visual stimulus instead of the inertial stimulus. However, this explanation doesn’t fully explain the findings as the bias was still less than the offset and the normal visual stimulus still had a larger bias than the inverted one.”

Minor requests for revision
4) Although the writing and English usage is in most cases standard, there is considerable room for improvement. Many small typos (e.g., in the Abstract "whish" should be "which") are evident throughout. Also English usage needs to be checked:

Thanks, this has been corrected.

p. 3: "they must share self-motion as the common causation" should be "they must be consistent with self-motion as the
common causation".

Done.

p. 3: "Perception of heading direction is an ecologically relevant situation..." should be something like "Perception of
heading direction is an ecologically relevant sensory-based ability..."

Done.

p. 3: "... been shown in the current laboratory:" should be "... been shown by our group:"

Done.

p. 3: "... (i.e. the perception of self-motion induced by a visual stimulus while stationary) should be "... (i.e. the illusory
perception of self-motion induced by a visual stimulus while the observer is stationary)

Done.

p. 4: "... and inertial motion seems required for ..." should be "... and inertial motion seems to be required for ..."

Done.

p. 5: "... disparity angle of visual ..." should be "... angular deviation of visual ..."

Done.

p. 7: "The subjects wore a helmet that was coupled to the motion platform while ..." The use of the verb "coupled" needs
to be explained here. Was head motion measured independently of platform motion and if so how?

The head motion was not measured independently from the platform motion in this study. We changed “coupled” to “attached”. It is also clarified: “In this study, the head movement was not measured separately from the platform as significant decoupling was felt to be unlikely for the type of motion used.”.

p.9: "... re-presented" should be "presented again ..."

Done.

p. 9: "... cumulative distribution function". Unclear if this distribution was normal (Gaussian) or otherwise.

This now clarified as “normal”.

p. 11: "... p. 3: "... in the current laboratory:" should be "... from our group:"

Done.

p. 11: "walking through blowing slow ..." should be "walking through blowing snow ..."

Done.

p. 11: "... but the influence of the inverted visual stimulus..." could be "... but the influence of the incongruent visual
stimulus..."

Done.

p. 11: "... so in most subjects the inverted visual stimulus biased inertial perception." could be "... so in most subjects even the inverted visual stimulus biased inertial perception."

Done.

p. 11: "average 64% of the normal one." could be "average 64% of the congruent one."

Done.

p. 12: "components that don’t match the visual stimulus." should be "components that are incongruent with those of the visual stimulus."

Done. Thank you for these detailed comments.

Reviewer #4: This paper investigates the role of visual motion profile on visual vestibular integration. The authors use two motion profiles one that is consistent with the velocity of the vestibular motion profile and one that is the inverted form of the profile. The paper is clearly written, and it is always nice to see the individual participant data.
I have some comments about the interpretation and analysis.

Thanks.

Comments:
Introduction/Discussion
The data shows that both motion profiles are integrated with the vestibular information as illustrated by the shift in the visual-vestibular PSE. Furthermore, the shift is less impacted by the inverted motion profile than the consistent motion profile. The authors posit that this is I'm the inverted motion profile is less it is integrated. While it is not unreasonable, another possibility is that there is a difference in the reliability of the responses between the two visual motion profiles, which was the case in Butler et al 2014, the raised cosine was more reliable than the constant velocity. This would mean that the inverted motion profile would be weighted less due to reliability and not causation. The authors could address this by analyzing the standard deviation (sigma) values of the fits of the cumulative distribution functions. If this cannot be done the authors should mention this possible caveat in the discussion.

The reviewer raises an excellent point which we had not previously considered. The standard deviations (sigma) of the psychometric function fits were measured but not previously reported. Overall, the normal stimulus had a sigma of 6.1 ± 0.4 (SEM) and the inverted stimulus had a sigma of 5.6 ± 0.4 (SEM). Although this suggests that the inverted stimulus may have been perceived as slightly more reliable this difference was not statistically significant (paired t-test, p = 0.4). Similarly, there were no differences in sigma based on the angular deviation of the visual stimulus. We have added Fig. 5 to report the sigma for each subject and a section of the Results and Discussion to address this. The paragraph in the Discussion states:

“It is well known that multisensory integration relies on the relative reliability of the stimuli[Acerbi, 2018 #2017][Butler, 2010 #1807][Knill, 2004 #858][Angelaki, 2009 #624][Fetsch, 2010 #1673]. The current findings could be potentially explained if the inverted visual stimulus was perceived as less reliable than the normal visual stimulus. Butler et al. found the raised cosine was more reliable than the constant velocity[Butler, 2015 #1854], although both were robustly integrated. In this study, the difference in the width (sigma) of the psychometric function fits between the two types of stimuli was not statistically significant (paired t-test, p=0.4). This suggests the perceived reliability of the stimuli did not explain the observed larger bias in the normal visual stimulus.”

It looks like there is no effect of visual offset (see below for suggested analysis). Were the authors surprised by this and could they write a line about it in the discussion.

Thanks. We have added a paragraph to the discussion to address this:

“The size of the visual offset in this study didn’t make a difference in either the size of the bias or the sigma of the psychometric function fit. Multiple offsets were included because one hypothesis was that common causation might be perceived at a larger offset if the visual stimulus was consistent with the acceleration profile, but this was not borne out in the data. The smallest offset tested was 45° which was large compared with the bias, and this may be why the bias did not depend on the size of the offset. The bias may reach some maximal value after which further visual offsets have no effect. We have also found that the reliability of the visual stimulus decreases as it gets more laterally displaced[Crane, 2012 #1280] so that larger displacements may have been counteracted by a lower perceived reliability. It is possible, if not likely, that smaller offsets would have produced a smaller bias, but this is outside of the scope of the current experiments.”

Analysis

The data were fit to a cumulative distribution function. If this function is Gaussian, please explicitly state this.

It is now clarified that this was “a normal (Gaussian) cumulative distribution function..”

The statistical analysis seems to be conducted in the wrong order; the ANOVA should be performed first, followed by t-tests as post hoc analyses. The appropriate ANOVA should be a 2 Motion Profile (Normal, Inverted) × 3 Offset (30, 45, 60) ANOVA. While it is mentioned that this analysis was conducted, the results concerning the offset are not discussed in the results section. Regardless of whether the results are significant, they are of interest and should be addressed. This analysis would allow for a discussion on both profile and offset. If feasible, this ANOVA should be conducted on both the PSE and the sigma of the fits.

Thanks for pointing this out. The order has been corrected in the methods and the ANOVA suggested is now presented. The only significant effect on bias was the visual stimulus type. This is now stated in the first paragraph of the Results:

“An ANOVA was performed using visual stimulus type (normal, inverted) and the three non-zero offsets (45, 60, 75°). This (ANOVA, 5 DF, F-ratio 1.02) revealed visual stimulus type to be the only statistically significant factor (p = 0.029) with no effect of offset (p = 0.87) or stimulus*offset (p = 0.99).”

This was also run for sigma: “When the potential effects of visual stimulus type and offset were examined there were no statistically significant effects (ANOVA, 5 DF, F-ratio 0.123).”

Results
To enhance clarity, it would be helpful to provide additional details about Figure 2 at the beginning of the Results section to better link the mean, bias, and PSE.

“Heading perception was biased in the direction of the offset visual stimulus and the mean Subject #17 was an outlier in that their bias towards visual stimulus (bias) and sigma were determined from individual responses(Fig. 2).”

In the second paragraph of the Results section, the ANOVA is mentioned. Could the authors clarify this point? Additionally, more information on the individual participant analysis would be appreciated, particularly regarding how the standard error of the mean (SEM) was calculated.

The SEM issue is now clarified in the figure caption: “Error bars represent ±1 SEM which was calculated across all visual offsets (±45, ±60, and ±75°) collected in that subject.”

Very minor comment:
there's a typo in the abstract; whish should be which.

Thanks, this has been corrected.

Finally, here are some papers that might be of interest to the authors that seemed relevant to this work

Butler, J. S., Smith, S. T., Campos, J. L., & Bülthoff, H. H. (2010). Bayesian integration of visual and vestibular signals for heading. Journal of vision, 10(11), 23-23.
Butler, J. S., Campos, J. L., Bülthoff, H. H., & Smith, S. T. (2011). The role of stereo vision in visual–vestibular integration. Seeing and perceiving, 24(5), 453-470.
Drugowitsch, J., DeAngelis, G. C., Klier, E. M., Angelaki, D. E., & Pouget, A. (2014). Optimal multisensory decision-making in a reaction-time task. Elife, 3, e03005.
Fetsch, C. R., Pouget, A., DeAngelis, G. C., & Angelaki, D. E. (2012). Neural correlates of reliability-based cue weighting during multisensory integration. Nature neuroscience, 15(1), 146-154.
Rideaux, R., Storrs, K. R., Maiello, G., & Welchman, A. E. (2021). How multisensory neurons solve causal inference. Proceedings of the National Academy of Sciences, 118(32), e2106235118.
Yakubovich, S., Israeli-Korn, S., Halperin, O., Yahalom, G., Hassin-Baer, S., & Zaidel, A. (2020). Visual self-motion cues are impaired yet overweighted during visual–vestibular integration in Parkinson’s disease. Brain communications, 2(1), fcaa035.
Zaidel, A., Goin-Kochel, R. P., & Angelaki, D. E. (2015). Self-motion perception in autism is compromised by visual noise but integrated optimally across multiple senses. Proceedings of the National Academy of Sciences, 112(20), 6461-6466.
Zhou, L., & Gu, Y. (2023). Cortical mechanisms of multisensory linear self-motion perception. Neuroscience Bulletin, 39(1), 125-137.

Thanks some of these references are now cited.
