# Supplementary material for: Effect of inverted visual acceleration profile on vestibular heading perception
Source: PLoS One. 2025 May 28;20(5):e0323348. doi: 10.1371/journal.pone.0323348 (PMC12118926; doi:10.1371/journal.pone.0323348)
Supplement: Attachment — Submitted filename: Revision 2 response to reviewer comments-1.docx [file pone.0323348.s006.docx]

Reviewer #2: The authors have thoroughly addressed all of my questions, and I have no further comments at this time.

**Response: Thanks**

Reviewer #3: The authors have responded to each of my queries. However, the revised text needs some editing by a native speaker of English with experience in scientific writing.  Minor points requiring final revision

1) p. 10: "In the current manuscript ..." should state " In the current study ..."

**Response: This was fixed accordingly**

2) p. 12 and elsewhere: "The width of the psychometric function ..." should state "The slope of the psychometric function ..."

**Response: This was fixed accordingly**

 3) p. 12 and elsewhere: "... significant effects (ANOVA, 5 DF, F-ratio 0.123)." should state "... significant effects (ANOVA, F = 0.123, df = 5)."

**Response: This was fixed accordingly**

4) p. 12: "Sigma was poorly correlated ..." should state "Sigma was not correlated ..."

**Response: This was fixed accordingly**

5) p. 14: "... we chose to leave it in." should either state "... we chose to leave him in." or rephrase the sentence as "An argument could be made for excluding data from this subject as statistical outliers but, we chose to leave them in."

**Response: This was fixed accordingly**

6) p. 15: "...(I,e. visual only or initial only)..." should state "..(I,e. visual only or inertial only)..."

**Response: This was fixed accordingly**

7) p. 16: "...in some previous studies in the current laboratory" should state ""...in previous studies from our group"

**Response: This was fixed accordingly**

8) p. 17: "...This effect was statistically significant (p < 0.0001 ). Thus,..." should state "...This effect was statistically significant (p < 0.0001 ), thus..."

**Response: This was fixed accordingly**

**Response: The authors thank the reviewer for what was clearly a careful reading of this revision. We have made the above changes as well as fixed other English grammar errors in the paper. The one exception was the initial two-way ANOVAs for mean and sigma that reviewer #4 had suggested changes to make more clear. Those are now presented in as a two-way ANOVA. In the previous version it was presented in a confusing way in which it was unclear if a one-way or two-way ANOVA was done.**

Reviewer #4: The authors have addressed most of my comments but I just want to clarify the ANOVA and how it has been reported. It is a bit confusing as in the analysis the ANOVA is given as an offset x stimulus 3(45 deg, 60 deg and 70 deg)x2(normal and inverted). But in the results it seems to be 6x1 (45 deg x normal, 60 deg x normal, 70 deg x normal, 45 deg x inverted, 60 deg x inverted and 70 deg x inverted). If it is a 2x3 the DoF should be: for the stimulus 1,19 for the offset 2,38 for the interaction 2,38 If it is a 6x1 then the DoF is 5 but it does not test the offset and stimulus separately, which means was a followup ANOVA conducted. Can the authors clarify this. What I would suggest is for the ANOVAs report the results as F(df1,df2)=?,p< and for the t-tests state t(df)=??,p

**Response: Thanks for noticing this and pointing it out. The ANOVA was initially done as a two-way ANOVA as the reviewer suggests, although it was presented in a confusing way. Using the stimulus type (normal, inverted) and non-zero offsets (45, 60, and 70) we did a two-way ANOVA with df = 1, df = 2. The ANOVA was repeated as a two-way ANOVA.**

**This is now made clear in the Methods: ”A two-way analysis of variance (ANOVA) was done using the three non-zero offsets (45, 60, and 75°) and the two visual stimulus types (normal and inverted).”**

**It is also made clear in the Results with regard to the effect on mean: “A two-way ANOVA was performed using visual stimulus type (normal, inverted) and the three non-zero offsets (45, 60, 75°). This (ANOVA, F(1,2) = (4.88, 0.14)) revealed that visual stimulus type is the only statistically significant factor (p = 0.028) with no effect of offset magnitude (p = 0.87).”**

**A similar statement was changed for the effect on sigma: “When the potential effects of visual stimulus type and offset were examined there were no statistically significant effects using a two-way ANOVA to look at stimulus type and non-zero offsets (ANOVA, F(1,2) = (0.30, 0.17)) revealed that neither stimulus type (p = 0.58) nor offset magnitude (p = 0.84) were significant factors. ”**
